# Patients on the Transplant Waiting List Have Anti-Swine Leukocyte Antigen Class I Antibodies

**DOI:** 10.4049/immunohorizons.2300056

**Published:** 2023-09-15

**Authors:** Zheng-Yu Wang, Luz Reyes, Jose Estrada, Christopher Burlak, Victor Novara Gennuso, Mosely O. Tector, Sam Ho, Matt Tector, A. Joseph Tector

**Affiliations:** * Department of Surgery, University of Miami School of Medicine, Miami, FL; † Makana Therapeutics, Miami, FL; ‡ Gift of Hope Organ and Tissue Donor Network, Itasca, IL

## Abstract

Organ supply remains inadequate to meet the needs of many patients who could benefit from allotransplantation. Xenotransplantation, the use of animals as organ donors, provides an opportunity to alleviate this challenge. Pigs are widely accepted as the ideal organ donor, but humans and nonhuman primates have strong humoral immune responses to porcine tissue. Although carbohydrate xenoantigens have been studied intensively, the primate Ab response also targets class I and class II swine leukocyte Ags (SLAs). Human Abs that recognize HLAs can cross-react with SLA molecules because epitopes can be shared across species. However, ∼15% of people may also exhibit Abs toward class II SLAs despite lacking Abs that also recognize class II HLAs. Here, we extend these studies to better understand human Ab responses toward class I SLAs. When tested against a panel of 18 unique class I SLA proteins, 14 of 52 sera samples collected from patients in need of an organ transplant contained Abs that bound class I SLAs. Class I SLA–reactive sera may contain IgM only, IgG, only, or IgM and IgG capable of recognizing the pig proteins. The presence of class I HLA–reactive Abs was not essential to generating anti–class I SLA Ig. Last, anti–class I SLA reactivity varied by serum; some recognized a single SLA allele, whereas others recognized multiple class I SLA proteins.

## Introduction

Results in xenotransplantation using pig kidneys in preclinical pig to nonhuman primate models is improving to the point where it is reasonable to consider implementing pilot trials in humans to eliminate the shortage of donor kidneys that has plagued kidney transplantation since its inception (1). The dominant cause of kidney loss in the first 6 months after transplant in our preclinical experience has been Ab-mediated rejection (2, 3). An obvious group of patients who will merit immediate consideration for inclusion in clinical trials is the highly HLA sensitized, for whom, because of the presence of anti-HLA Abs, an immunologically compatible human donor kidney with a suitable pretransplant crossmatch cannot be found. The question whether the swine leukocyte Ags (SLAs) are xenoantigens continues to evolve, and its answer is a critical prerequisite to moving forward with successful clinical trials (4–7).

SLA is the porcine MHC and consists of both class I and class II Ags. SLAs and HLAs share many similarities, but several important differences also exist. Classical class I SLA genes (SLA-1, SLA-2, SLA-3) code for 45 kDa transmembrane glycoproteins with three extracellular domains, α1, α2, and α3, that associate noncovalently with β_2_-microglobulin (reviewed in 8). The SLA I alleles are named numerically to indicate that they share greater sequence homology with each other than with any other species class I MHC molecule (9). Classical class I SLA cell surface expression follows the general pattern of SLA 2 > SLA 1 > SLA 3 (8).

The high density of expression of class I MHC molecules makes these molecules important Ags to evaluate in the early phases of post–kidney transplant survival. Donor-specific Abs (DSAs) to class I Ags typically result in allograft loss in the first 6 mo after transplant, and, if present at high enough levels, they cause immediate graft failure via hyperacute rejection (10–12). Class II MHC Ags are of lower density and expressed primarily on professional APCs and selective tissue types (e.g., normal renal microvascular endothelium). Consequently, class II DSAs result in an indolent form of graft loss characterized by glomerulopathy and thrombotic microangiopathy occurring over many months (13–16). Evaluation of prospective recipients for anti–SLA I Abs is critical to initial renal xenograft trials because of the potential for these Abs to cause early graft loss. Once some degree of clinical success is achieved, then working toward better class II SLA compatibility may be feasible.

Previously, we have created class I MHC knockout (KO) pigs and cells to demonstrate that SLA-1, SLA-2, and SLA-3 can be targeted by human Abs (6). However, these studies focused on patients having Abs toward class I HLA molecules that could drive cross-reactivity with the homologous SLA proteins. These studies did not indicate whether patients lacking Abs to class I HLA could have anti-class I SLA Abs. This report had two goals: (1) to produce a simple cell expression system enabling the study of class I SLA proteins as possible xenoantigens and (2) to determine whether a patient could produce class I SLA Abs in the absence of class I HLA humoral immunity. To address these goals, we created an HLA-deficient human cell line that can express single alleles of class I SLAs. Eighteen versions of this cell line, each expressing a unique class I SLA allele, were used to evaluate 52 patients on the transplant waitlist for the presence of Abs to each SLA molecule. Our results demonstrate that 27% (14 of 52) of patients had Abs present that bound to SLA I alleles. In addition, Abs toward class I SLAs were found even in patients who lacked class I HLA humoral reactivity.

## Materials and Methods

### Human sera

Human sera samples were remnants of clinical samples being discarded from the clinical laboratory. The samples and their associated calculated panel reactive Ab (cPRA) as a measure of allosensitization were obtained under a protocol approved by the University of Miami Institutional Review Board.

### Development of engineered C1R cells expressing single SLA class I

C1R, a human B lymphoblastoid cell line, was genetically engineered using the gRNA and Cas9 nuclease system as described previously (6). The engineered C1R (e-C1R) lacked multiple surface molecules (HLA class I and class II, Fc receptor, and IgG), which cause background binding of Abs in human sera. Next, class I SLA/HLA allele e-C1R was created using pREP4 mammalian expression vector (Fisher Scientific, V004-50) encoding the following open reading frames: SLA-1*07:02, SLA-1*07:05, SLA-1*08:05, SLA-1*11:02, SLA-1*12:01, SLA-1*13:01, SLA-1*18:01; SLA-2*02:02, SLA-2*03:02, SLA-2*05:01, SLA-2*05:02, SLA-2*06:01, SLA-2*07:01, SLA-2*10:01, SLA-2*12:02; SLA-3*04:02, SLA-3*05:02, and SLA-3*06:02, as well as HLA-A*02:03 and HLA-A*03:01 alleles. Cells demonstrating increased cell surface β_2_-microglobulin expression after transfection were sorted and maintained with hygromycin antibiotics at 200 μg/ml (Invitrogen). SLA open reading frame cDNA sequences were obtained from the IMGT MHC database (17).

### Flow cytometry

The following Abs were used: anti-pig SLA class I (clone JM1E3, Invitrogen); anti–β_2_-microglobulin Ab (clone 2M2; BioLegend, San Diego, CA); anti-human HLA-DR, DP, DQ Ab (clone Tü39; BioLegend); and anti-human CD32 (FcγRII) Ab (clone FUN-2; BioLegend). Flow cytometric analysis of sorted cells was performed using an Accuri C6 flow cytometer.

### Flow cytometric crossmatch assay

To assess the levels of cross-reactive Abs in patient sera, a flow cytometric crossmatch assay was performed as described previously (18). Briefly, patient sera in 1:4 dilution were tested against allele C1R (total 52 sera samples); Alexa Fluor 488 goat anti-human IgG (catalog no. 109-546-097, Jackson ImmunoResearch Laboratories Inc.) or Alexa Fluor 488 goat anti-human IgM (catalog no. 109-546-129, Jackson ImmunoResearch Laboratories Inc.) were used as the secondary Abs. Flow cytometric data were acquired using a BD FACSLyric flow cytometer. Being a cell line consisting of a single cell type, a simple gating strategy using forward scatter (FSC) and side scatter, followed by comparing FSC height versus FSC area to identify singlet cells, was used to define the population of single cells being analyzed. Flow cytometry files were analyzed in FlowJo version 10 (BD Biosciences, Ashland, OR). Because of the limited sample volume typically available for clinically discarded samples, it was possible to assay each sample only once.

### Statistical analyses

Graph and data analyses were completed using Prism 9 for Macintosh (GraphPad Software, La Jolla, CA).

### Class I SLA protein sequence analysis

Identification of amino acids that may contribute to SLA epitopes was accomplished by retrieving the relevant SLA sequences obtained from the IMGT MHC database and comparing the sequences of SLA proteins recognized by Abs from human serum versus the SLA proteins that failed to react with the human Abs in that same serum (17). The BLAST algorithm was used to verify identical and different amino acids at each position of relevant SLA molecules (19).

## Results

### Description of e-C1R single SLA class I–expressing reagents

**FIGURE 1.**
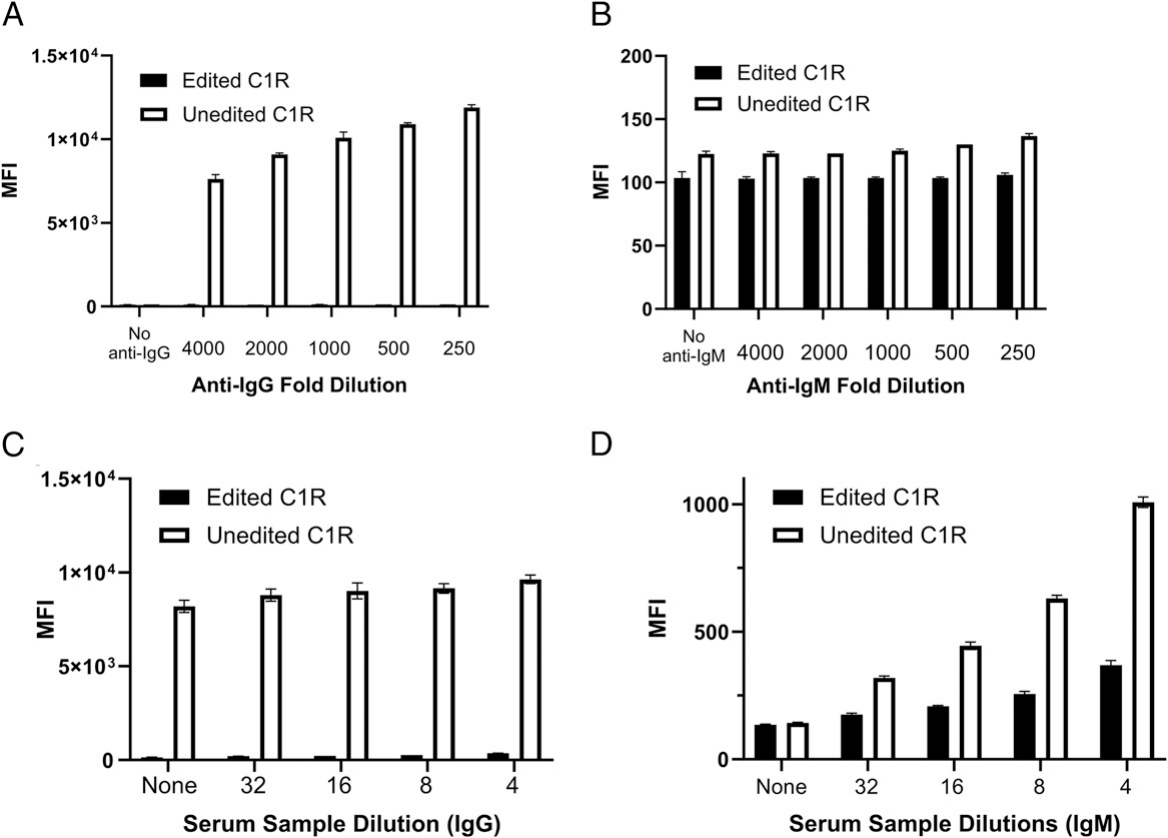
Producing an ideal cell line to facilitate the detection of human Abs specific for class I SLA proteins. Being of human B-cell origin, C1R cells express several molecules that may generate irrelevant signals in crossmatch assays. Gene editing of a human C1R B lymphoblastoid cell line to inactivate endogenous genes encoding class I and class II HLA, IgG, and an Fc receptor reduced this unwanted signal. (**A**) Fluorescent anti-human IgG binds unedited C1R cells at high levels (white bars). This signal was markedly reduced in the edited C1R cells (black bars). (**B**) Fluorescent anti-human IgM binds unedited C1R cells poorly (white bars), and edited C1R cells had little additional reduction in IgM signal. (**C** and **D**) A collection of pooled human sera were incubated at different titrations with the edited and SLA-deficient C1R cells (black bars) and unedited C1R cells (white bars). Binding to edited C1R cells was diminished compared with unedited cells.

**FIGURE 2.**
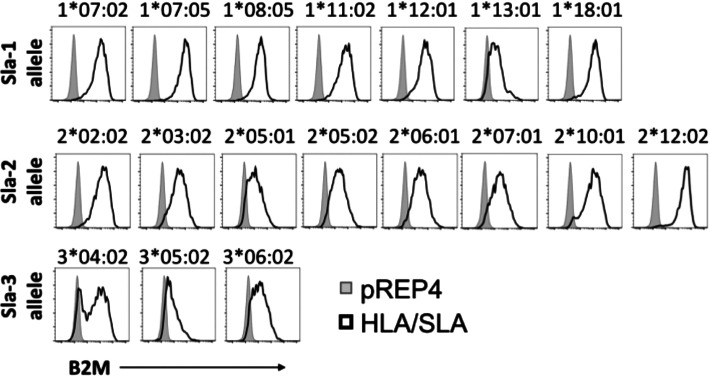
Expressing class I SLA in edited C1R cells. The gene-edited C1R cells were transfected with individual open reading frames to encode 18 SLA class I molecules, respectively, for Sla-1 (7 alleles), Sla-2 (8 alleles), and Sla-3 (3 alleles). The pREP4 vector–alone transfected cells were used as a control. Cell surface expression of the class I HLA and SLA proteins was assessed by flow cytometry using an anti–β_2_-microglobulin (B2M) Ab. Representative histograms are shown for individual allele C1R cells (black line) compared with pREP4 alone (gray histograms).

Each SLA allele was expressed in a human B lymphoblastoid cell line engineered to eliminate endogenous targets of human Abs (HLA molecules and Fc receptors) and Ab detection reagents (IgG proteins). Editing endogenous genes in C1R eliminated their background reactivity with the secondary reagents that detect human Ig (Fig. 1A, 1B) and with human serum Abs (Fig. 1C, 1D). Like HLA, to reach the cell surface, class I SLA binds to β_2_-microglobulin, a protein that lacks variability. Consequently, a β_2_-microglobulin mAb recognizes the same target on every SLA molecule and can be used to measure relative expression of each class I SLA protein complex. Although the cell surface abundance of different SLA proteins varied, all were easily distinguishable from the negative control cell signal containing the expression vector that lacked a cDNA for SLA (Fig. 2). Table I shows pig haplotypes known to contain each of 18 evaluated class I SLA alleles.

**Table I.** Amino acid residues common to antigenic SLA but absent from nonantigenic SLA

Serum	IgM	IgG	Potential Epitope Location
A		147, 157	α2
D	23, 24, 56, 107, 206		α1, α2
E	97, 148, 151		α2
F		176, 177, 183	α2, α3
G		73	α1
H		176, 177, 183	α2, α3
J	12, 55, 56, 58, 80		α1
L		9, 11, 21, 66, 213	α1, α3
M		9, 11, 21, 66, 213	α1, α3
N	9, 11, 21, 66, 213		α1, α3

Primary amino acid sequences from antigenic and nonantigenic class I SLA as determined for each serum (A–N) were compared. Amino acids found in only the antigenic sequences but absent from nonantigenic sequences are noted, by position, as likely candidates to make up an epitope. The domain of the class I SLA protein (α1, α2, α3) where the amino acids reside are also noted.

### Binding of Abs from transplant candidates to SLA proteins

**FIGURE 3.**
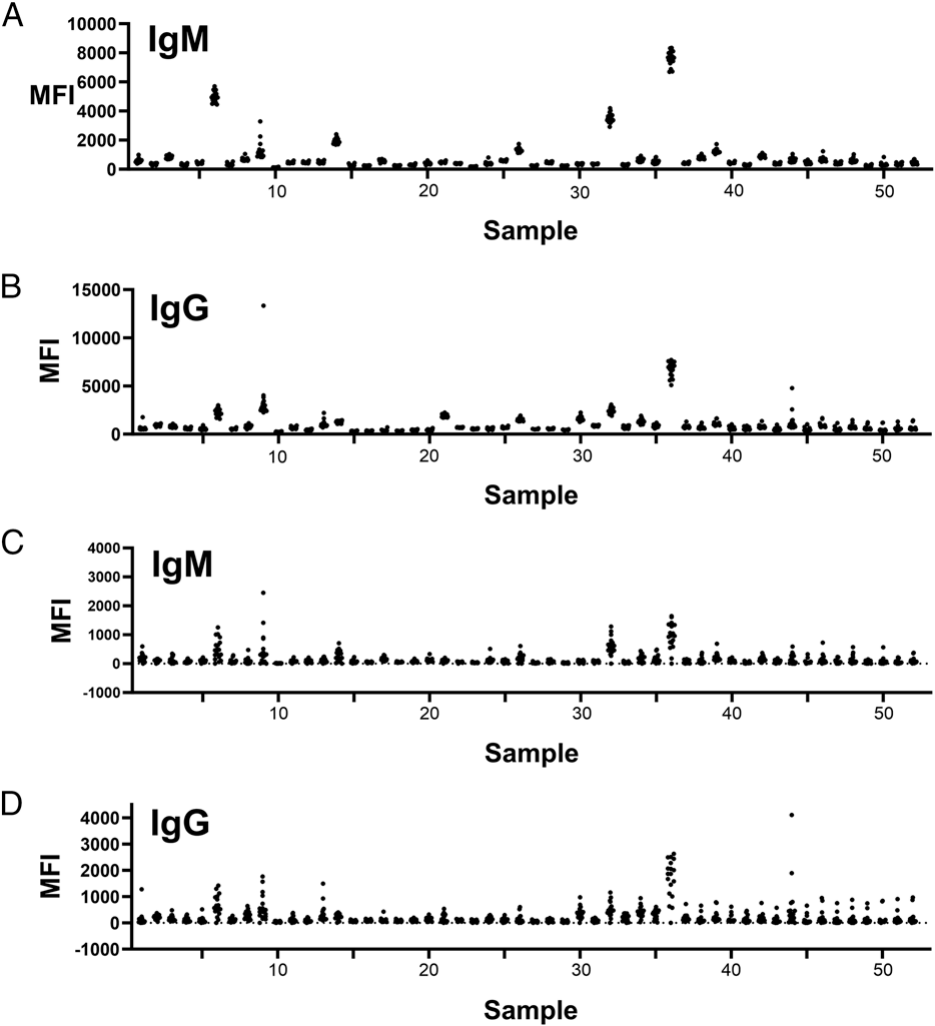
Evaluation of human IgM and IgG binding to cell lines expressing individual alleles of class I SLA. Serum (*x*-axes) from 52 patients on the transplant waitlist were incubated with 18 class I SLA–positive cell lines described in Figs. 1 and 2. The vector-only cell line lacking SLA expression was also tested. IgM and IgG binding to each line was detected by flow cytometry, and MFIs are reported (*y*-axes). Raw MFIs for IgM (**A**) and IgG (**B**) binding to each cell line are shown. For each serum tested in (**C**) (IgM) and (**D**) (IgG), MFI from the least reactive cell line was subtracted from itself (zero values) and the remaining cell lines.

Sera from 52 patients being considered for allotransplant were incubated with each of the 18 SLA-expressing cell lines and the vector-only control cell line. Fluorescent anti-human IgM and IgG secondary Abs were added to detect bound Ig. Fig. 3A (IgM) and Fig. 3B (IgG) show raw median fluorescence intensities (MFIs) for each of 52 sera against each SLA protein. These results demonstrate that some sera have markedly elevated background against all cell lines (as examples in Fig. 3A, sera samples 6, 9, 14, 21, 26, 30, 32, and 36). Our past analyses of serum Ab binding to SLA proteins compared ratios of MFI when a serum sample was incubated with a cell line expressing SLA versus the same cell line when devoid of SLA (5, 6, 20). Typically, we used 10-fold greater MFI of Abs on a cell expressing SLA compared with a negative control cell as a cutoff to indicate binding to the SLA protein. However, in samples with elevated background signal, the ratio of SLA reactivity versus a negative control decreases, which minimizes the detection sensitivity. To avoid this problem, we combined two approaches in the present study. The first involved analyzing each serum against every cell line and subtracting the lowest MFI value from all results for that sample. Fig. 3C and 3D show this analysis for IgM and IgG, respectively. Although we included the edited and SLA-deficient C1R cell as a negative control, some allele–serum combinations yielded results where this negative control generated higher fluorescence than a cell line expressing an individual SLA protein. We assumed that any allele–serum combination exhibiting less fluorescence than the vector-only cell reflected no Ab binding to that SLA protein. Consequently, we subtracted the background of the least fluorescent cell line, whether it was the true negative vector-only cell or an SLA-expressing cell, to ensure there were no negative values when subtracting background. The SLA-deficient cell fluorescence values were not included in the mean and SD calculation. Positive Ab–SLA interactions were chosen to have an MFI value that was at least 2 SD above the group mean for each allele.

**FIGURE 4.**
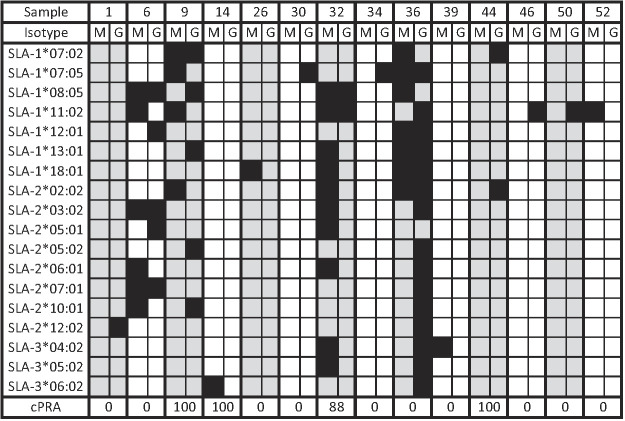
Summary of human IgM and IgG binding to class I SLA proteins. Fourteen of the 52 human sera examined in Fig. 3 showed IgM or IgG reactivity with at least one SLA-expressing cell line. Black squares indicate serum–allele combinations that produced a positive binding result. The remaining 38 sera demonstrated neither IgM nor IgG reactivity with any SLA protein. The bottom row indicates the cPRA calculated for each serum to indicate if each serum contains or lacks anti-HLA Abs.

The above analysis identified a variety of SLA reactivity patterns in 14 of the 52 sera samples (Fig. 4). Many of these sera samples showed limited reactivity, recognizing only a single SLA protein (sera samples 1, 14, 26, 30, 34, 39, 46, 50, and 52), whereas others demonstrated reactivity with multiple alleles (sera samples 6, 9, 32, 36, and 44). As indicated by the cPRA score in the bottom row of Fig. 4, some patients contained anti–class I HLA Abs (cPRA >0), whereas others did not (cPRA 0). SLA binding was not limited to a single Ig isotype, because both IgG and IgM showed reactivity. Sequence comparisons of antigenic and nonantigenic SLA proteins were performed to identify potential residues that may contribute to a xenoepitope. In 10 of the 14 sera samples showing SLA reactivity, amino acids could be identified that were common to every antigenic SLA, but not in any nonantigenic proteins (Table II). These residues were in all three extracellular domains of the SLA protein (α1, α2, α3), suggesting that any portion of the class I protein may contribute to xenoantigenicity.

**Table II.** Analysis of SLA allele frequency in known haplotypes

Alleles	Haplotypes
SLA-1*07:02	32.0
SLA-1*07:05	50.0
SLA-1*08:05	6.0
SLA-1*11:02	56.0
SLA-1*12:01	35.0
SLA-1*13:01	35.0
SLA-1*18:01	57.0
SLA-2*02:02	73.0
SLA-2*03:02	10.0
SLA-2*05:01	11.0
SLA-2*05:02	7.0
SLA-2*06:01	9.0
SLA-2*07:01	25.0
SLA-2*10:01	35.0
SLA-2*12:02	NA
SLA-3*04:02	73.0, 32.0
SLA-3*05:02	12.0, 26.0, 35.0, 39.0, 40.0, 60.0, 64.0, 69.0, 72.0, 79.0
SLA-3*06:02	16.0, 19.0

The haplotypes containing each known SLA allele studied in this report are indicated.

## Discussion

Avoiding Ab-mediated rejection is key to achieving long-term success in kidney xenotransplantation (21). Disrupting carbohydrate-modifying genes in pigs eliminates glycan-based xenoantigens on pigs. Other xenoantigens, the SLAs, are proteins. Unlike carbohydrate xenoantigens, which are identical among all pigs, the highly polymorphic SLA molecules vary from animal to animal. In a limited study of Ab binding to class I SLA proteins, we showed that a patient’s anti-SLA Abs might recognize one SLA protein over another. The goal of this study was to expand upon these initial results and determine how frequently patients awaiting kidney transplants may produce Abs against class I SLA proteins. Therefore, we evaluated human Ab binding to 18 unique class I SLA proteins. Using cells engineered to express a single SLA molecule, we previously showed that Ig arising from exposure to HLA could produce Abs that cross-reacted with SLAs (4, 6). However, identifying anti–class I SLA IgG and IgM in people with panel reactive Ab equal to 0% demonstrates that allosensitization is not a prerequisite to their formation.

Using gene editing tools and cellular flow cytometry assays, we can evaluate specific anti–SLA I and anti–SLA II molecules as xenoantigens (4–6, 20). Our results shed light on key questions for the development and implementation of clinical xenotransplantation. Here we find that 27% (14 of 52) of randomly selected waitlisted patients had anti–SLA I DSAs. In allotransplantation, when patients have anti-HLA humoral immunity, they are paired with donors selected to lack expression of HLA proteins targeted by the recipient’s Abs. It may be possible to apply the same approach in xenotransplantation when attempting to avoid SLA Abs (i.e., screen multiple pigs to avoid the specific anti-SLA Abs a recipient may have). As a first evaluation of how effective this approach may be, we determined how many SLA haplotypes contained each of the 18 alleles we studied (Table I). Abs toward SLA-3*05:02 would likely limit the pig donor pool the most because they are present in the greatest number of pig haplotypes.

The ultimate solution to these problems may be handled by using genome editing of the SLA to eliminate the entire SLA I or alter amino acids to eliminate epitopes. The utility of this strategy has been confirmed in vitro for SLA class II and could eliminate an antigenic determinant with only minor alteration of the SLA I in a donor pig (20). The elimination of an amino acid–based epitope could produce a donor pig with a still robust SLA I repertoire and cause minimal disruption to the donor pig immune system before its use as a renal donor. A more extensive approach that might be necessary for recipients who have broadly SLA I–reactive DSA would be to create donor pigs with classical SLA I deleted. Class I SLA–deficient pigs have been created (22, 23). We created an animal with exon 4 in the α3 domain disrupted so that there is no β_2_-microglobulin binding site for the classical SLA I molecules (17). This genetic engineering strategy is appealing because it does not affect the β_2_-microglobulin molecule, which has other important functions in the pig and its kidney other than simply associating with SLA I molecules. Pigs with classical class I SLA molecules and GGTA1 deletions have been created, and their kidneys have been transplanted into rhesus monkeys. Prolonged survival of GGTA1/SLA I KO pig kidneys was achieved, and the pathobiology of rejection was minimally altered compared with SLA I–replete GGTA1/KO pig kidneys (21). More recently, pigs have been created with GGTA1/CMAH/β4GalNt2 KO in addition to β_2_-microglobulin deletion (23). These pigs were healthy, and they have deletion of nonclassical in addition to classical SLA I alleles. It is unknown whether the nonclassical SLA I alleles play a role in renal xenograft rejection, but it will be important to evaluate whether nonclassical SLA I Ag are in fact relevant xenoantigens.

Combining our study with work evaluating class II SLA as a xenoantigen suggests that anti-SLA Abs will be an important initial consideration for many patients being considered as candidates for xenotransplantation trials. For xenotransplantation to be successful in the near term, we will need to accept this fact and develop the ability to evaluate which patients do and do not have anti-SLA Abs so that we can avoid early preventable graft loss/failure.
